# Annual acknowledgement of manuscript reviewers

**DOI:** 10.1186/s12938-015-0001-0

**Published:** 2015-06-25

**Authors:** Kenneth R Foster, Fong-Chin Su

**Affiliations:** Department of Bioengineering, University of Pennsylvania, Philadelphia, USA; Medical Device Innovation Center, National Cheng Kung University, Tainan City, Taiwan

## Abstract

The Editors of *BioMedical Engineering OnLine* would like to thank all the Reviewers who have contributed to the journal in Volume 13 (2014).

**Vahid Abdollah**

Canada

**Eduardo Abreu**

United States of America

**U Rajendra Acharya**

Singapore

**Ted Acott**

United States of America

**Arti Ahluwalia**

Italy

**Iftikhar Ahmad**

Finland

**Moiz Ahmad**

United States of America

**Rufai Ahmad**

Nigeria

**Ceyhun Burak Akgül**

Turkey

**S. Hamed Alavi**

United States of America

**Mark Albert**

United States of America

**Clif Alferness**

United States of America

**Mehdi Alilou**

Belarus

**Javier Alonso**

Spain

**David Amarantini**

France

**Rouzbeh Amini**

United States of America

**Gholamreza Aminian**

Iran

**Samreen Amir**

Pakistan

**Kurt Ammer**

Austria

**Pokpong Amornvit**

Thailand

**Kai Keng Ang**

Singapore

**Javier Mauricio Antelis Ortiz**

Mexico

**Maria Rosa Antognazza**

Italy

**Konstantinos Antypas**

Norway

**Daisuke Anzai**

Japan

**Nooranida Arifin**

Malaysia

**Mina Arvin**

Netherlands

**Alberto Avolio**

Australia

**Danilo Babin**

Belgium

**James Baish**

United States of America

**Peña Baquedano**

Spain

**Jerome Baron**

Brazil

**Teodiano Bastos-Filho**

Brazil

**Sebastian Baumgaertel**

United States of America

**Noah Bedard**

United States of America

**Michael Bentley**

United States of America

**Balazs Benyo**

Hungary

**Matthew Berryman**

Australia

**Martijn Beudel**

Netherlands

**Pinaki Bhattacharya**

Belgium

**Iwona Bialik-Okopniarek**

Poland

**Joseph Bidwell**

United States of America

**Fetsje Bijma**

Netherlands

**Mehmet Bilgen**

Turkey

**Maria Cristina Bisi**

Italy

**Maria Paula Bonomini**

Argentina

**Richard Bostelmann**

Germany

**Stuart Bowyer**

United Kingdom

**Patrick Boyle**

United States of America

**Andrew Bradley**

Australia

**Neil Bruce**

Mexico

**Christoph Brueser**

Germany

**Amanda Buck**

United States of America

**Wojciech Budzianowski**

Poland

**Sarah Buehler**

Germany

**Ivo Bukovsky**

Czech Republic

**Vladimír Bures**

Czech Republic

**Kevin Burrage**

Australia

**Giovanni Calcagnini**

Italy

**Jason Carey**

Canada

**Donato Cascio**

Italy

**Vincent Casey**

Ireland

**Branko Celler**

Australia

**Benedetta Cesqui**

Italy

**Adrian Chan**

Canada

**David Chapman**

United States of America

**Geoff Chase**

New Zealand

**Raul Chavez Santiago**

Norway

**Yen-Wei Chen**

Japan

**Hui Chen**

China

**Chung-Ming Chen**

Taiwan

**Gin-Shin Chen**

Taiwan

**Jyh-Cheng Chen**

Taiwan

**Shixiong Chen**

China

**Weidong Chen**

China

**Weng-Pin Chen**

Taiwan

**Teddy Cheng**

Australia

**Vamsy Chodavarapu**

Canada

**Kian Jon Chua**

Singapore

**Edward Ciaccio**

United States of America

**A. Don Clark**

United States of America

**Chris Clinard**

United States of America

**Claudio Cobelli**

Italy

**Michele Conti**

Italy

**Christine Curcio**

United States of America

**Krzysztof Cyran**

Poland

**Adson da Rocha**

Brazil

**Mehmet Dalli**

Turkey

**Siedlecki Damian**

Poland

**Alessander Danna-dos-Santos**

United States of America

**Rafael Davalos**

United States of America

**Philip de Chazal**

Australia

**Gianluca De Santis**

Belgium

**Stefan Debener**

Germany

**Ricard Delgado-Gonzalo**

Switzerland

**Xiaoyan Deng**

China

**Zhi-De Deng**

United States of America

**Valérie DEPLANO**

France

**Thomas Desaive**

Belgium

**Matteo D'Este**

Switzerland

**Marco Di Rienzo**

Italy

**Alejandro Diaz-Sanchez**

Mexico

**Michael Doellinger**

Germany

**Enqing Dong**

China

**Fangfang Dong**

China

**Shiying Dong**

Australia

**Ivan Dotsinsky**

Bulgaria

**Andreas Drauschke**

Austria

**Bin Duan**

United States of America

**Qi Duan**

United States of America

**Leila Eadie**

United Kingdom

**Ayman El-Baz**

United States of America

**Mohamed Elgendi**

Canada

**Deniz Erbulut**

Turkey

**Carles Escera**

Spain

**Samuel Ewing**

United Kingdom

**Alessandra Fabbro**

Italy

**Qianjin Feng**

China

**Jose M Ferrandez**

Spain

**Stefan Fischer**

Germany

**Brian Flynn**

United Kingdom

**Juan Fontana**

Argentina

**Domenico Formica**

Italy

**Kenneth Foster**

United States of America

**Anderson Freitas**

Brazil

**Daniel Fried**

United States of America

**Monique Frize**

Canada

**Jian Fu**

China

**Nicholas Gaddum**

United Kingdom

**Humberto Gamba**

Brazil

**Tapan Gandhi**

United States of America

**Hang Gao**

Belgium

**Xiaohong Gao**

United Kingdom

**Gonzalo A. Garcia**

Spain

**Amit Gefen**

Israel

**Dhanjoo Ghista**

United States of America

**kaiser Giri**

India

**Luis Paulo Gomes Mascarenhas**

Brazil

**Cihun-Siyong Gong**

Taiwan

**He Gong**

China

**Qin Gong**

China

**Haiyan Gong**

United States of America

**Joshua Guag**

United States of America

**Lan-Yuen Guo**

Taiwan

**Taeyiung Ha**

Korea, South

**Ibrahim Habli**

United Kingdom

**W. David Hairston**

United States of America

**Neda Haj-Hosseini**

Sweden

**Ryan Halter**

United States of America

**Niels Hammer**

Germany

**Hai-Chao Han**

United States of America

**Chengzong Han**

United States of America

**Jae Yong Han**

Germany

**Jingjia Han**

United States of America

**Yael Hanein**

Israel

**Hari Hariharan**

United States of America

**Prasannna Hariharan**

United States of America

**Kamran Hassani**

Iran

**Jens Haueisen**

Germany

**Jan Havlik**

Czech Republic

**Michel Heijnen**

United States of America

**Thordur Helgason**

Iceland

**Ralph Herkert**

United States of America

**Thomas Heyse**

Germany

**Kathleen Hickey**

United States of America

**Alain Hillion**

France

**Kai-Yu Ho**

United States of America

**David Holder**

United Kingdom

**Ales Holobar**

Slovenia

**Joerg Holstein**

Germany

**Eun-Kyoung Hong**

United States of America

**Tim Howells**

Sweden

**Tzu-Chien Hsiao**

Taiwan

**Yi-Zeng Hsieh**

Taiwan

**Ming-Lun Hsu**

Taiwan

**Po-Lin Hsu**

China

**Dewen Hu**

China

**Jinlian Hu**

Hong Kong

**Chih-Chung Huang**

Taiwan

**Feng Huang**

China

**Haw-Ming Huang**

Taiwan

**Clark Hung**

United States of America

**Dietmar Werner Hutmacher**

Australia

**Changmo Hwang**

Korea, South

**Jae Y. Hwang**

Korea, South

**ahmed idhammad**

Morocco

**Takeshi Ikuma**

United States of America

**Nilesh Ingle**

United States of America

**Clara Ionescu**

Belgium

**Zafer Iscan**

United States of America

**Cinthia Itiki**

Brazil

**Richard Jaspers**

Netherlands

**Nadeem Javaid**

Pakistan

**Venugopal Jayarama Reddy**

Singapore

**Gwanggil Jeon**

Korea, South

**Ning Jiang**

Germany

**Philippe Jouvet**

Canada

**Michael Joy**

Canada

**Ming Ju**

Taiwan

**Muammar Kabir**

United States of America

**Kaihua Zhang**

Hong Kong

**Sei-ichiro Kamata**

Japan

**Rita Kandel**

Canada

**Bart Kaptein**

Netherlands

**Bilge Karacali**

Turkey

**Tahir Karaman**

Turkey

**Karim S Karim**

Canada

**Henryk Kasprzak**

Poland

**Ulrich Katscher**

Germany

**Peter Kazanzides**

United States of America

**Mohsen Kazemi**

Malaysia

**Ahamed Khan**

Malaysia

**Shihab Khogali**

United Kingdom

**Bee Ee Khoo**

Malaysia

**Youngho Kim**

Korea, South

**Tatsuya Kin**

Canada

**Harald Kirchsteiger**

Austria

**Hiroko Kitaoka**

Japan

**Ching-Chang Ko**

United States of America

**Jih-Yang Ko**

Taiwan

**Seokbum Ko**

Canada

**Shohta Kodama**

Japan

**Petra Kok**

Netherlands

**Kranthi Kolli**

United States of America

**Grzegorz Konieczny**

Poland

**Robert Koprowski**

Poland

**Anna Korzynska**

Poland

**Yordan Kostov**

United States of America

**Tanja Kraus**

Austria

**Victor Krauthamer**

United States of America

**Ashnil Kumar**

Australia

**Nicholas Kurniawan**

Netherlands

**Oh-Kyong Kwon**

Korea, South

**David Labbe**

Canada

**Martin Lawitzky**

Germany

**Jesús Lázaro**

Spain

**Yeon Soo Lee**

Korea, South

**Christopher Lee**

United States of America

**Jinseok Lee**

Korea, South

**Hyung-Min Lee**

United States of America

**Kyoung Joung Lee**

Korea, South

**Minho Lee**

Korea, South

**Soo Yeol Lee**

Korea, South

**Tzer-Min Lee**

Taiwan

**Jianbo Lei**

China

**Edward Lemaire**

Canada

**Bing Nan Li**

China

**Haiyun Li**

China

**Huiqi Li**

China

**John Li**

United States of America

**Junbo Li**

China

**Kefeng Li**

United States of America

**Jie Li**

United States of America

**Liang Li**

China

**Mao LI**

Australia

**Qing Li**

Australia

**Qingli Li**

China

**Jianyi Li**

China

**Lun-De Liao**

Singapore

**Jun Liao**

United States of America

**Ian Liau**

Taiwan

**Dieter Liepsch**

Germany

**Chun-Li Lin Lin**

Taiwan

**Chun-Pin Lin**

Taiwan

**Jiun-hung Lin**

Taiwan

**Chien-Ju Lin**

Taiwan

**Ting-Sheng Lin**

Taiwan

**Biyue Liu**

United States of America

**Jianli Liu**

China

**Jie Liu**

China

**Dongxu Liu**

China

**Huafeng Liu**

China

**Jian Liu**

United States of America

**Perng-Ru Liu**

United States of America

**Gerald Loeb**

United States of America

**Nicolas Lomenie**

France

**Alejandro Lopez Valdes**

Ireland

**Edmond Lou**

Canada

**Wajid Aziz Loun**

Pakistan

**Albert Lozano**

United States of America

**Qijin Lu**

United States of America

**Leonidas Lymberopoulos**

Greece

**Ching-Hou Ma**

Taiwan

**Jianhua Ma**

China

**Warren Macdonald**

United Kingdom

**Jarmo Malinen**

Finland

**Robert Malkin**

United States of America

**Maria-Cristina Manzanares-Céspedes**

Spain

**Francois MArchal**

France

**Joan Marti**

Spain

**Fermín Martínez Solís**

Mexico

**Sergio Martinez-Chapa**

Mexico

**David Mas**

Spain

**Mohd Hanafi Mat Som**

Japan

**Eugenio Mattei**

Italy

**Dave Meaney**

United States of America

**Paolo Melillo**

Italy

**Sabato Mellone**

Italy

**Claudia Mello-Thoms**

Australia

**Silvio Mario Meloni**

Italy

**Geoffrey Meltzner**

United States of America

**Arianna Menciassi**

Italy

**Frerk Meyer**

Germany

**Matjaz Mihelj**

Slovenia

**Joseph Miller**

United States of America

**Shogo Minagi**

Japan

**Gradimir Misevic**

Switzerland

**Lino Misoguti**

Brazil

**Mehran Moazen**

United Kingdom

**Knut Möller**

Germany

**Niyousha Mortaza**

Canada

**Seyed Mojtaba Mousavi**

Malaysia

**Richard Muench**

Germany

**Subhas Mukhopadhyay**

New Zealand

**Joung H. Mun**

Korea, South

**Chi-Woong Mun**

Korea, South

**Alessio Murgia**

Netherlands

**Kheline Naves**

Brazil

**Karina Needham**

Australia

**Jonathan Newell**

United States of America

**G. Andre Ng**

United Kingdom

**Tetsuya Nishimoto**

Japan

**Domen Novak**

Switzerland

**Wioletta Nowak**

Poland

**Anna Nowinska**

Poland

**Anna Nowinska**

Poland

**Syam Nukavarapu**

United States of America

**Eiichiro Okumura**

Japan

**Nick Oliver**

United Kingdom

**Stig Ollmar**

Sweden

**Michael Orendurff**

United States of America

**Mariusz Oszust**

Poland

**Darryl Overby**

United Kingdom

**Andrei Pakhomov**

United States of America

**Pertti Palo**

United Kingdom

**Dorin Panescu**

United States of America

**Davide Paolino**

Italy

**Theodore Papaioannou**

Greece

**Jeremy Patterson**

United States of America

**Eugenio Pedullà**

Italy

**Hanchuan Peng**

United States of America

**Daniel Peterson**

United States of America

**Lacomme Philippe**

France

**Yangjun Pi**

China

**Kinga Pielichowska**

Poland

**Ulrike Pielmeier**

Denmark

**Farzin Piltan**

Iran

**Weeraphat Pon-On**

Thailand

**Punit Prakash**

United States of America

**Sergey Pravdin**

Belgium

**Philip Procter**

France

**Shouliang Qi**

China

**Xin Qi**

United States of America

**Aike Qiao**

China

**Han Qin**

United States of America

**Alberto Rainoldi**

Italy

**Suhrud Rajguru**

United States of America

**Jayakumar Rangasamy**

India

**Marwan Refaat**

United States of America

**Gernot Reishofer**

Austria

**Sabrina Renzi**

Italy

**Rodolfo Repetto**

Italy

**Alex Reyzelman**

United States of America

**Denise Ricieri**

Brazil

**Isabel Rodriguez**

Spain

**Yonetsu Ryo**

Japan

**Umberto Sabatini**

Italy

**Hassan Saeedi**

Iran

**Zahra Safaeepour**

Iran

**Yoshifumi Saijo**

Japan

**Solomon Samuel**

United States of America

**Niklas Sandler**

Finland

**Saeid Sanei**

United Kingdom

**M Iqbal Saripan**

Malaysia

**Bernd Saugel**

Germany

**James Saunders**

United States of America

**Stephen Sawiak**

United Kingdom

**Alexander Schmidt-Richberg**

Germany

**Galen Schneider**

United States of America

**Cedric Schwartz**

Belgium

**Ervin Sejdic**

United States of America

**Jin Keun Seo**

Korea, South

**Fabrizio Sergi**

Italy

**Maxime Sermesant**

France

**Igor Sersa**

Slovenia

**Salvatore Sessa**

Japan

**Amir Akramin Shafie**

Malaysia

**Ding-Lan Shen**

Taiwan

**Hackjoon Shim**

Korea, South

**Vladimir Shin**

Korea, South

**Guofa Shou**

United States of America

**Ian Sigal**

United States of America

**Kim Sivesgaard**

Denmark

**Andrzej Skowron**

Poland

**Laura Slane**

United States of America

**David Sliney**

United States of America

**Robert Smith**

Australia

**Witold Sokolowski**

United States of America

**Pengfei Song**

United States of America

**Abdullah Ruhi Soylu**

Turkey

**Fredrik Ståhl**

Sweden

**Stergios Stergiou**

United States of America

**Berend Stoel**

Netherlands

**Fong-Chin Su**

Taiwan

**Steven Su**

Australia

**Kaiqiong Sun**

China

**Ming Sun**

United States of America

**Grazyna Swierczek-Zieba**

Poland

**Abigail Swillens**

Belgium

**Montadar Taher**

Malaysia

**Ta-Wei Tai**

Taiwan

**Yohei Takai**

Japan

**Jen Hong Tan**

Singapore

**Tomohiko Tanaka**

Japan

**Shuo Tang**

Canada

**Rongbiao Tang**

China

**Shih-Tsang Tang**

Taiwan

**Zhongzhao Teng**

United Kingdom

**Slawomir Teper**

Poland

**Yuji Teramura**

Japan

**Lan Tian**

China

**Godfrey Town**

United Kingdom

**Bram Trachet**

Switzerland

**Maciej Tulodziecki**

Poland

**Paul Turner**

United States of America

**Kadriye Tuzlakoglu**

Turkey

**Virgilio Valente**

United Kingdom

**Max Valentinuzzi**

Argentina

**Ricardo Vardasca**

Portugal

**Georgios Varsos**

United Kingdom

**Jelle Veraart**

United States of America

**Brigitte Von Rechenberg**

Switzerland

**Frank Vrionis**

United States of America

**Eric Walker**

United States of America

**Baikun Wan**

China

**Honggang Wang**

United States of America

**Li Wang**

United States of America

**Tianyi Wang**

United States of America

**Tzu-Wei Wang**

Taiwan

**Lei Wang**

China

**Xintong Wang**

United States of America

**Yuan-Fang Wang**

United States of America

**Yijun Wang**

United States of America

**Slawomir Wilczynski**

Poland

**Kwok Chuen Wong**

Hong Kong

**Milosz Wozniczko**

Poland

**Dalei Wu**

United States of America

**Josh Wu**

Taiwan

**Shuicai Wu**

China

**Lei Xi**

United States of America

**Yuxiang Xing**

China

**Jingjing Xu**

United States of America

**Wenwei Xu**

United States of America

**Lisheng Xu**

China

**Fei Yan**

China

**Tai-Hua Yang**

United States of America

**Yu-Sheng Yang**

Taiwan

**Junjie Yao**

United States of America

**Saami Yazdani**

United States of America

**Mete Yeginer**

Turkey

**Ming-Long Yeh**

Taiwan

**Erwei Yin**

China

**Yuichi Yoshii**

Japan

**Jian-hong Yu**

Taiwan

**Xiao-Hua Yu**

United States of America

**Jie Yuan**

China

**Ming Yuchi**

China

**Ning Yue**

United States of America

**Yonghyeon Yun**

Korea, South

**Matej Zajc**

Slovenia

**Rad Zdero**

Canada

**Guanqun Zhang**

United States of America

**Guangming Zhang**

United States of America

**Ming Zhang**

Canada

**Tinggang Zhang**

United States of America

**Yangsong Zhang**

China

**Yan Zhang**

United Kingdom

**Yingchun Zhang**

United States of America

**Aili Zhang**

China

**Zhanqi Zhao**

Germany

**Guyoan Zheng**

Switzerland

**Wei Zheng**

China

**Jianlong Zhou**

Australia

**Ping Zhou**

United States of America

**Shoujun Zhou**

China

**Xiaoming Zhou**

China

**Zhongxing Zhou**

China

**Yufang Zhu**

China

**Christian Zweifel**

Switzerland

